# Novel Characteristics of Mitochondrial Electron Transport Chain from *Eimeria tenella*

**DOI:** 10.3390/genes10010029

**Published:** 2019-01-08

**Authors:** Makoto Matsubayashi, Daniel Ken Inaoka, Keisuke Komatsuya, Takeshi Hatta, Fumiya Kawahara, Kimitoshi Sakamoto, Kenji Hikosaka, Junya Yamagishi, Kazumi Sasai, Tomoo Shiba, Shigeharu Harada, Naotoshi Tsuji, Kiyoshi Kita

**Affiliations:** 1 Division of Veterinary Science, Graduate School of Life and Environmental Sciences, Osaka Prefecture University, 1-58, Rinku Orai Kita, Izumisano, Osaka 598-8531, Japan; ksasai@vet.osakafu-u.ac.jp; 2 School of Tropical Medicine and Global Health, Nagasaki University, 1-12-4, Sakamoto, Nagasaki 852-8523, Japan; kitak@nagasaki-u.ac.jp; 3 Department of Host-Defense Biochemistry, Institute of Tropical Medicine (NEKKEN), Nagasaki University, 1-12-4, Sakamoto, Nagasaki 852-8523, Japan; 4 Department of Biomedical Chemistry, Graduate School of Medicine, The University of Tokyo, 7-3-1, Hongo, Bunkyo-ku, Tokyo 113-0033, Japan; keisuke-kiyose@kcf.biglobe.ne.jp; 5 Department of Parasitology, Kitasato University School of Medicine, 1-15-1, Kitasato, Minami-ku, Sagamihara, Kanagawa 252-0374, Japan; htakeshi@med.kitasato-u.ac.jp (T.H.); tsujin@med.kitasato-u.ac.jp (N.T.); 6 Mocky Poultry Practice, Shinmeidai 2-5-33-810, Hamura-shi, Tokyo 205-0023, Japan; funuwo@f7.dion.ne.jp; 7 Department of Biochemistry and Molecular Biology, Faculty of Agriculture and Life Science, Hirosaki University, Aomori 036-8561, Japan; sakamok@hirosaki-u.ac.jp; 8 Department of Infection and Host Defense, Graduate School of Medicine, Chiba University, 1-8-1, Inohana, Chuo-ku, Chiba 260-8670, Japan; hikosaka@chiba-u.jp; 9 Research Center for Zoonosis Control, Hokkaido University, North 20, West 10, Kita-ku, Sapporo, Hokkaido 001-0020, Japan; junya@czc.hokudai.ac.jp; 10 Department of Applied Biology, Graduate School of Science Technology, Kyoto Institute of Technology, Matsugasaki, Sakyo-ku, Kyoto 606-8585, Japan; tshiba@kit.ac.jp (T.S.); harada@kit.ac.jp (S.H.)

**Keywords:** Coccidium, apicomplexa, *Eimeria tenella*, membrane protein, mitochondria, electron transport chain, succinate dehydrogenase, ubiquinone, inhibitor

## Abstract

*Eimeria tenella* is an intracellular apicomplexan parasite, which infects cecal epithelial cells from chickens and causes hemorrhagic diarrhea and eventual death. We have previously reported the comparative RNA sequence analysis of the *E. tenella* sporozoite stage between virulent and precocious strains and showed that the expression of several genes involved in mitochondrial electron transport chain (ETC), such as type II NADH dehydrogenase (NDH-2), complex II (succinate:quinone oxidoreductase), malate:quinone oxidoreductase (MQO), and glycerol-3-phosphate dehydrogenase (G3PDH), were upregulated in virulent strain. To study *E. tenella* mitochondrial ETC in detail, we developed a reproducible method for preparation of mitochondria-rich fraction from sporozoites, which maintained high specific activities of dehydrogenases, such as NDH-2 followed by G3PDH, MQO, complex II, and dihydroorotate dehydrogenase (DHODH). Of particular importance, we showed that *E. tenella* sporozoite mitochondria possess an intrinsic ability to perform fumarate respiration (via complex II) in addition to the classical oxygen respiration (via complexes III and IV). Further analysis by high-resolution clear native electrophoresis, activity staining, and nano-liquid chromatography tandem-mass spectrometry (nano-LC-MS/MS) provided evidence of a mitochondrial complex II-III-IV supercomplex. Our analysis suggests that complex II from *E. tenella* has biochemical features distinct to known orthologues and is a potential target for the development of new anticoccidian drugs.

## 1. Introduction

Poultry coccidiosis is an intestinal disease caused by infection of the apicomplexan protozoan parasites from the genus, *Eimeria*. These parasites produce high economic losses because of decreased chicken growth performance and expensive control systems against the disease. Due to the coccidiosis, the annual worldwide loss in poultry industries, including egg production, was estimated at up to two billion dollars [1]. Amongst the seven *Eimeria* spp. that infect chicken, *Eimeria tenella* have been recognized as the most pathogenic parasite due to its high mortality rate following infection. So far, the control of coccidiosis is depending on a variety of anticoccidial agents or live attenuated vaccines. However, these preventive therapies possess severe restricted use in chickens and difficulties to achieve this complete prevention to *Eimeria* infection. For instant, the routine use of prophylactic medications is not allowed in chicken farms because of the serious problems raised to both the animals’ health and chicken product safety. In addition, the administration of live attenuated vaccines to chickens requires re-infection to achieve adequate immunity [2]. Therefore, the development of new cost-effective drugs to carry out satisfactory prevention is currently needed.

In infected chickens, the disruption of the tissue by the *Eimeria* parasite growth leads to chronic or acute, and watery or bloody diarrhea. The parasites of *Eimeria* spp. possess a complex life cycle in infected chickens, by initiating the oral ingestion of the sporulated oocysts, including two sporozoites within four sporocysts. The sporozoites released in the intestinal tract invade into the mucosa, and then, undergo several asexual (generating merozoites within schizonts) and sexual stage (generating micro- and macro-gametocytes) developments, resulting in the formation of oocysts [3]. Eventually, these noninfectious oocysts released in the feces begin to sporulate, which is triggered by environmental factors, such as temperature, humidity, and oxygen concentration, to acquire the infectivity [4]. In contrast to the sporulation development outside the host, the asexual and sexual proliferations proceed under low oxygen concentrations in the intestines [5], and thus, it is believed that the *Eimeria* parasites have evolved specific mechanisms in order to survive and grow within the specialized host microenvironments. However, the molecular basis of the adaptation to the host environment remains unknown.

In intestinal helminth parasites, such as *Ascaris suum*, *Haemonchus contortus*, and *Echinococcus multilocularis*, the mitochondria play key role in adaptation to the host environment by drastically changing the electron transport chain (ETC) composition according to each life cycle stage [6,7,8,9]. For instance, the ETC of egg and L3 larvae stages of *A. suum* have a similar composition to the one from the mammalian host and is composed by respiratory complexes I, II, III, and IV and is dependent on electron carriers, which are ubiquinone and cytochrome *c*, and oxygen as the final electron acceptor by a well-known process named oxygen respiration [6,10]. However, because of the low concentration of oxygen in the host intestine, the ETC from the adult stage of *A. suum* switch to anaerobic respiration known as fumarate respiration, which is composed by complex I coupled with the reverse reaction of complex II and a low potential quinone specie, named rhodoquinone [6]. In fumarate respiration, fumarate is used as the final electron acceptor producing succinate, which is excreted as the end product [11]. In other apicomplexan parasites, such as *Plasmodium falciparum* or *Toxoplasma gondii*, which are closely related parasite to *Eimeria* spp., the parasite’s mitochondria are also key organelles involved in cellular metabolic pathways essential to parasite’s adaptation and survival within different hosts’ environment. Amongst the mitochondrial metabolic pathways, the ETC is believed to be the key pathway for energy generation, pyrimidine de novo biosynthesis, and purine salvage pathways (through fumarate cycle), at least in *P. falciparum* [12,13,14]. In addition to ubiquinone, a low potential quinone species involved in anaerobic respiration, menaquinone, has been detected in *P. falciparum* [15]. Due to the indispensability of ETC, it is indeed a promising therapeutic target pathway not only against helminthes, but also protozoan parasites [6,7,16].

In contrast to changes in mitochondria morphology according to the *P. falciparum* life cycle stages, the mitochondria of *E. tenella* is observed as elongated structures throughout its life cycle [17]. According to the genome sequence of *E. tenella*, five ETC dehydrogenase which can utilize oxidized quinone (ubiquinone) as an electron acceptor are conserved: The non-proton motive type II NADH (nicotinamide adenine dinucleotide) dehydrogenase (NDH-2), succinate dehydrogenase (SDH, Complex II), malate:quinone oxidoreductase (MQO), glycerol-3-phosphate (G3P) dehydrogenase (G3PDH), and dihydroorotate (DHO) dehydrogenase (DHODH) [18]. We have previously identified by RNA sequencing analysis that the expression of NDH-2, SDH, MQO, and G3PDH are upregulated in *E. tenella* at the stages of sporulating and/or excysted sporozoites [19], which deserved a detailed biochemical study. Despite the recognition of these enzymes as potential targets for the development of new chemotherapeutic agents against parasitic diseases, to the best of our knowledge, information about the mitochondria of *E. tenella*, including the methods for preparation of mitochondria-rich fraction or the activities of the ETC enzymes in detail, are not available.

Here, to determine the biochemical feature of *E. tenella* ETC enzymes, we have developed a new method for the preparation of mitochondria-rich fraction from *E. tenella* sporozoites and conducted for the first time, a comprehensive biochemical study of the ETC enzymes.

## 2. Materials and Methods

### 2.1. Sequence Analysis

The sequence analysis of each ETC enzymes deduced from the gene sequence were performed using the ClustalWS platform (https://www.genome.jp/tools-bin/clustalw) and the amino acid percentage identity calculated by the pairwise alignment tool from Jalview 2.9.0b2 software (http://www.jalview.org/), according to the developer’s protocol [20].

### 2.2. Preparations of Eimeria tenella Oocysts and Sporozoitess

The NIAH strain of *E. tenella*, which was maintained by passage in 2- to 3-week-old chicks (Nisseiken, Tokyo, Japan) at the Laboratory of Parasitic Diseases, National Institute of Animal Health (Tsukuba, Ibaraki, Japan), was used in this study. Chicks were housed in wire-floored cages in coccidian-free rooms and had free access to feed and water that contained no anticoccidial drugs or antibiotics. The animals were treated in accordance with protocols approved by the Animal Care and Use Committee, NIAH (Approval Nos. 11-026, 12-029). Chicks were orally inoculated with 2 × 10^4^ mature oocysts, and feces were collected after 6–8 days. Immature oocysts were purified from feces by using the sugar flotation method [21], incubated for 2–3 days at 28 °C in 2.5% (w/v) potassium dichromate (Wako, Osaka, Japan) to promote sporulation, and then stored at 4 °C for 2–3 months before use.

For purification of sporozoites, sporulated oocysts (3 × 10^8^) were centrifuged at 1500× *g* for 5 min at 4 °C and washed with phosphate-buffered saline (PBS). The oocysts were further incubated in 8.5%–13.5% (w/v) sodium hypochlorite for 20 min at 4 °C and centrifuged at 1500× *g* for 5 min at 4 °C. The sodium hypochlorite treatment was repeated twice, followed by washing with PBS for three times. After the last centrifugation, parasites were incubated in 4 mL of excystation medium [0.25% (w/v) trypsin (Merck, Darmstadt, Germany) and 1% (w/v) taurodeoxycholic acid (Sigma, St. Louis, MO, USA) in Hanks’ balanced salt solution (Sigma), pH 7.4], at 4 °C for overnight. Oocysts were centrifuged at 1750× *g* for 10 min at 4 °C, suspended in 1 mL of PBS. Next, 1 mL of 0.5–0.7 mm glass beads (As one, Osaka, Japan) was added and the oocysts were broken by vortexing for approximately 2.5 min at room temperature until >95% of the oocysts were broken, releasing the sporocysts, under a microscopic observation. Ten milliliters of PBS were added, mixed briefly, and the parasites were collected by retrieving the supernatant. This step was repeated four times. Collected parasites were centrifuged at 1400× *g* for 10 min at 4 °C, re-suspended in 20 mL of excystation medium, and incubated in a water bath shaker for 2 h at 41 °C with vigorous shaking of 160 rpm. After adding PBS up to 50 mL, parasites, including excysted sporozoites, were collected by centrifugation at 1750× *g* for 10 min at room temperature. Next, the pellet was washed once in 50 mL of PBS. After centrifugation at 1750× *g*, 10 min at room temperature, the parasites were suspended in 20 mL PBS and filtered by a passage in a membrane filter of 20 µm (Steriflip, Merck Millipore, Darmstadt, Germany). The filter was washed by an additional 80 mL of PBS, and the parasites were concentrated by centrifugation at 1750× *g*, 10 min at room temperature. The parasites were suspended in 25 mL of PBS and centrifuged at 100× *g* for 2 min at 4 °C. The supernatant, containing the sporozoites, was collected and kept on ice. The remaining pellet was resuspended in 25 mL of PBS centrifuged again at 100× *g* for 2 min at 4 °C. The supernatant was collected and combined to the supernatant from the previous step. A total of 50 mL of combined supernatant was centrifuged at 1800× *g* for 10 min twice, and the pellets consisting of sporozoites (approximately 3 × 10^8^) were resuspended in 12.5 mL of MSE buffer (210 mM mannitol, 10 mM sucrose, 1 mM disodium EDTA (ethylenediaminetetraacetic acid), and 50 mM Tris-HCl, pH 7.5) containing 10 mM disodium malonate and protease inhibitor cocktail (Roche Diagnostics, Tokyo, Japan). The parasite suspension was centrifuged at 1800× *g* for 10 min at 4 °C. The final pellet was suspended in 5 mL of MSE buffer.

### 2.3. Transmission Electron Microscopy (TEM) of Eimeria tenella

To morphologically confirm the mitochondria of *E. tenella*, the sporozoites prepared as described above were fixed with 2% (w/v) glutaraldehyde (Electron Microscopy Sciences, Hatfield, PA, USA) and 2% (w/v) paraformaldehyde (PFA, TAAB laboratories Equipment, Berks, UK) in 0.1 M phosphate buffer (PB, pH 7.4) at room temperature for 2 h and kept at 4 °C overnight. The cells were washed with 0.1 M PB (pH 7.4) containing 0.1 M sucrose (Wako, Osaka, Japan), post-fixed with 1% (w/v) OsO4 in 0.1 M PB containing 0.1 M sucrose at 4 °C for 2 h, dehydrated in an ethanol series, and embedded in Epon resin (TAAB Laboratories Equipment). Ultra-thin sections were stained with saturated uranyl acetate (TAAB Laboratories Equipment) in distilled water for 20 min and Reynold’s lead citrate for 3 min and examined with an electron microscope (H-7500, Hitachi, Tokyo, Japan).

### 2.4. Preparation of Eimeria tenella Mitochondria-rich Fraction

The mitochondria-rich fraction of *E. tenella* sporozoites were obtained by two different cell disruption protocols. The purified sporozoites were disrupted using (i) the POLYTRON homogenizer (POLYTRON^®^ PT1200E, Central Scientific Commerce, Tokyo, Japan) at 70% of the maximum output by a cycle consisting of 2 min disruption and 5 min on ice for two cycles; or (ii) N_2_ cavitation (4639 Cell disruption Bomb, Parr, Moline, IL, USA) at 2000 psi for 20 min at 4 °C. Unbroken cells and cell debris were removed by centrifugation at 700× *g* for 10 min at 4 °C, and the supernatant was centrifuged at 30,000× *g* for 30 min at 4 °C. The mitochondria-rich fraction was recovered as the precipitate, which was suspended in MSE containing 10 mM disodium malonate and protease inhibitor cocktail. The protein concentration was determined according to the method of Lowry using bovine serum albumin as a standard [22].

### 2.5. Enzyme Assays

All enzyme assays were performed using 5–10 µg of the mitochondria-rich fraction using black quartz cuvettes in 0.6 mL reaction mixture at 25 °C. The reagents used in each assay were mixed with a reaction buffer containing 50 mM potassium phosphate (pH 7.4) (KPi Buffer) and 2 mM EDTA.

The NADH oxidase activity (NDH-2-Complex III-IV) was assay by the addition of 0.2 mM NADH to the mixture and following the decrease in absorbance of NADH at 340 nm (ε = 6.2 mM^−1^ cm^−1^) with UV-3000 double wavelength spectrophotometer (Shimadzu Corp., Kyoto, Japan).

Cytochrome *c*–linked NADH, G3P, malate, succinate, and DHO dehydrogenase activities were determined in a reaction mixture containing 30 µM horse heart cytochrome *c* and 2 mM KCN at 550 nm (ε = 19 mM^−1^ cm^−1^) by adding 0.2 mM NADH, 5 mM malate, 5 mM G3P, 8.3 mM succinate, and 0.4 mM DHO, respectively, into the reaction buffer.

Individual G3PDH, MQO, SDH, and DHODH activities were assayed at 278 nm in the presence of each substrate as described above, however, instead of cytochrome *c*, 50 µM of decylubiquinone (dUQ) (ε = 15 mM^−1^ cm^−1^) was added. The NDH-2 activity was measured following the consumption of NADH at 340 nm.

### 2.6. Analysis of the Quinone from Eimeria tenella

Quinone contents were analyzed by liquid chromatography-mass spectrometry (LC-MS) and the high-performance liquid chromatography equipped with photodiode array detector (HPLC-PDA) systems. To the 30 mg wet pellet of the *E. tenella* sporozoites, 600 μL of 2-propanol was added to extract quinones and quinols. To the 600 μL of supernatant, 6 μL of 2-propanol supplemented with 120 mM FeCl_3_ was added to oxidize all the quinol to the quinone [23]. After the removal of insoluble debris by filtration through a 0.2 μm pore-size filter (Ultra-free, millipore), 4 μL of the filtrated extracts was injected to the LC-MS system, consisting of a pump (L-2100, Hitachi, Japan), column oven at 40 °C (L-2300, Hitachi), analytical column (Inertsil ODS-3, 2 μm, 2.1 mm i.d. × 50 mm, GL Science, Tokyo, Japan), UV detector at 275 nm (L-2420, Hitachi), and ESI-TOF MS (NanoFrontier LD, Hitachi). The mobile phase used in our experiment was methanol/2-propanol/water (45/48/7, v/v/v), 0.1% (v/v) formic acid at a flow rate of 0.2 mL/min. To analyze with the HPLC-PDA system, 600 μL of oxidized extract was dried under nitrogen gas flow. The concentrated solution was obtained by adding 30 μL of 2-propanol and filtrated as described above. The HPLC-PDA system consisting of a pump (L-7610, Hitachi), column oven at 40 °C (L-7300, Hitachi), analytical column (Inertsil ODS-3, 2 μm, 2.1 mm i.d. × 50 mm, GL Science), and PDA detector (MD-4017, Jasco, Tokyo, Japan) was operated under the same mobile phase as the LC-MS analysis. As standard samples, menaquinone-7 (MK-7) (Wako, Osaka, Japan), ubiquinone-9 (UQ-9) (Sigma, St. Louis, MO, USA), and UQ-10 (Wako) were used. For UQ-8, MK-8, and demethylmenaquinone-8 (DMK-8), *Escherichia coli* HB101 extract was prepared essentially following the procedure described above for *E. tenella* quinone extraction.

### 2.7. Enzyme Inhibition Assay of Eimeria tenella Succinate Dehydrogenase

The 50% inhibitory concentration (IC_50_) values against succinate-cytochrome *c* reductase activity (Complex II-III) of the parasite mitochondria was determined varying the concentration of four compounds, which included atpenin A5 and carboxin, which are known as inhibitors for bovine complex II with the IC_50_ values of 0.004 and 1 μM, respectively [24]; siccanin, which was reported as an inhibitor of complex II from *Leishmania tarentolae*, *L. donovani*, *Trypanosoma brucei*, and *T. cruzi* with IC_50_ value of 0.190 µM, 1.17 µM, 0.368 µM, and 1.48 µM, respectively [25,26]; and flutolanil, which was reported as a potent and specific inhibitor of *Ascaris suum* complex II with an IC_50_ value of 0.081 μM [27]. As the positive control for complex II-III inhibition assay, 0.1 μM of stigmatellin (complex III inhibitor) was used [28].

### 2.8. High Resolution Clear Native Electrophoresis (HrCNE) and Succinate Dehydrogenase Activity Staining

The mitochondria-rich fraction of *E. tenella* sporozoites prepared as described above were incubated on ice for 10 min in 50 mM Tris-HCl pH 8.0, 10% (v/v) glycerol and 0.5% (w/v) sucrose monolaurate (SML). The solubilized mitochondrial membrane proteins were centrifuged at 200,000× *g* for 15 min at 4 °C. The resulting supernatant was mixed with final concentrations of 0.01% (w/v) Ponceau S in 10 mM Tris–HCl, pH 7.5 (Invitrogen, Carlsbad, CA, USA), 5% (v/v) glycerol, and loaded onto Novex 4%–16% (w/v) Bis–Tris gels (Invitrogen). HrCNE was performed in NativePAGE^TM^ running buffer (Invitrogen) supplemented with 0.02% (w/v) dodecylmaltoside and 0.05% (w/v) sodium deoxycholate at 150 V for 1 h, and then increased to 250 V under voltage constant at 4 °C. After the HrCNE, the gels were stained either by Coomassie brilliant blue (CBB) dye or subjected to SDH activity staining.

The SDH activity staining of mitochondrial ETC proteins was conducted by the modified methods as in a previous report [26]. After the HrCNE, the gels were washed twice in 5 mM Tris-HCl buffer (pH 7.4) for 5 min to remove the detergents from the running buffer. The equilibrated gel was soaked in 5 mM Tris-HCl buffer (pH 7.4) containing 2.5 mg/mL nitro blue tetrazolium chloride at room temperature for 5 min. Next, succinate and phenazine methosulfate were added at a final concentration of 20 mM and 4 mg/mL, respectively, and kept static in the dark. After the SDH stained band was clearly visible, the reaction was stopped by washing the gel several times with pure water.

### 2.9. Two Dimentional Sodium Dodecyl Sulfate Polyacrylamide Gel Electrophoresis (2D-SDS-PAGE) and Nano- Liquid Chromatography Tandem-Mass Spectrometry (LC MS/MS) Analysis of Eimeria tenella Succinate Dehydrogenase Stained Band

The SDH activity staining band was cut out from the HrCNE gels, soaked in 30 μL of 1 × SDS-PAGE sample buffer for 5 min, and loaded onto 12.5% (w/v) SDS-PAGE gel. The gels were stained by CBB for the visualization of proteins composing the SDH stained band. Additionally, the SDH activity stained band was sent for analysis by nano-LC-MS/MS (FUJIFILM Wako Pure Chemical Corporation). Identified protein fragment sequences were searched in the *E. tenella* genome database (http://protists.ensembl.org) using the MASCOT software (Matrix Science, Tokyo, Japan).

## 3. Results

### 3.1. Electron Transport Chain Dehydrogenases from Eimeria tenella

Five ETC dehydrogenases are conserved in the genome of *E. tenella*, which are the NDH-2, Complex II, MQO, G3PDH, and DHODH (Figures S1–S5). NDH-2 (Figure S1) is an enzyme with activity equivalent to respiratory complex I, but devoid of proton-pump activity and is required to re-oxidize NADH generated by mitochondrial pathways, such as the tricarboxylic acid (TCA) cycle [29]. Complex II is the only membrane bound enzyme from the TCA cycle in mammals and catalyzes the electron transfer from succinate to quinone (SQR) or from reduced quinol to fumarate (QFR) [7,24,26,30]. In mammals, complex II is composed by flavoprotein (SDH1, Figure S2), iron-sulfur cluster protein (SDH2, Figure S3), cytochrome *b* large (SDH3), and small subunits (SDH4). The SDH1 and SDH2 are well conserved in prokaryotes and eukaryotes (Figures S2 and S3) while the membrane anchor subunits formed by SDH3 and SDH4 are diverse [6]. MQO is a membrane protein found among bacteria kingdom and some protozoa (Figure S4) and functions as a member of the TCA cycle. Higher eukaryotes do not possess MQO, but instead, they possess the NAD^+^-dependent malate dehydrogenase, a soluble enzyme located in the mitochondria matrix [13,14,31]. G3PDH catalyzes the irreversible oxidation of G3P into dihydroxyacetone phosphate using ubiquinone as the electron acceptor and is well conserved among prokaryotes and eukaryotes (Figure S5). It is an enzyme involved in glycerol metabolism as well as gluconeogenesis [32,33]. Finally, DHODH catalyzes the oxidation of dihydroorotate into orotate with concomitant reduction of ubiquinone [34]. DHODH is the rate-limiting step in the pyrimidine de novo biosynthesis pathway and is required to generate UMP, the building block of DNA and RNA necessary to sustain life [35,36]. NDH-2 and MQO are not conserved in the animal kingdom and are considered as potential drug targets for the development of new antibiotics, and antifungal and antiparasitic agents [13,14,29].

### 3.2. Transmission Electron Microscopy Analysis of Eimeria tenella

**Figure 1:**
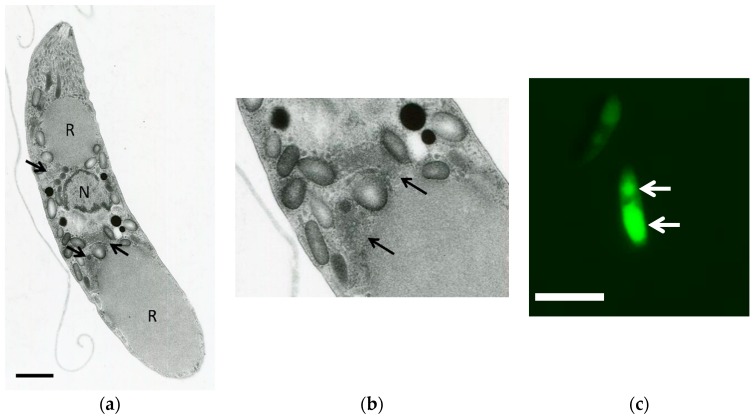
(**a**) Electron microscopy of *Eimeria tenella* sporozoite; (**b**) high magnification of the mitochondria of the parasite; (**c**) staining of sporozoites by mitochondrial specific probes (MitoTracker^®^, Thermo Fisher Scientific). Bars = 2 μm (**a**) and 5 μm (**c**). The positions of the mitochondria are indicated by the arrows. R: refractile bodies. N: nucleous.

The mitochondria within the sporozoites were morphologically confirmed by TEM. As shown in Figure 1a,b, the mitochondria from *E. tenella* possessing tubular cristae were observed around the anterior and posterior refractile bodies. In addition, the mitochondria from sporozoites could be stained by MitoTracker^®^ (Thermo Fisher Scientific, Waltham, MA, USA) (Figure 1c) and were observed surrounding the entire refractile body, which was consistent with the TEM analysis.

### 3.3. Preparation of Mitochondria-Rich Fraction

To perform biochemical study of *E. tenella* mitochondrial ETC enzymes, we evaluated the efficiency of the methods for preparation of mitochondria-rich fraction by two different cell disruptions protocols (Polytron or N_2_ cavitation) from *E. tenella* sporozoites. Also, the storage conditions that maintain stable mitochondrial NADH-cyt *c* and succinate-cyt *c* activities. As a result, the mitochondria-rich fraction obtained from sporozoites disrupted by the Polytron method exhibited a lower total activity for both activities than those disrupted by the N_2_ cavitation method (Table 1). When the mitochondria-rich fraction was stored at 4 °C for 48 h, both activities were reduced independently to the disruption method. However, when the mitochondria-rich fraction stored at −80 °C for 48 h, the NADH-cyt *c* and succinate-cyt *c* activities remained at 100% and 65% for the Polytron method and 74% and 87% for the N_2_ cavitation method, respectively. Therefore, we determined the specific activity of *E. tenella* ETC enzymes on the same day as the mitochondria-rich fraction isolation. For inhibition studies, the mitochondria-rich fraction stored at −80 °C was used within 3–4 days due to the higher yield of both activities compared to the Polytron method. By this protocol, we could obtain approximately 1.0–1.5 mg of mitochondria-rich fraction starting from 3 × 10^8^ sporozoites.

### 3.4. Characterization of Eimeria tenella Mitochondrial Electron Transport Chain.

Next, the specific enzyme activities of the mitochondrial respiratory chain from *E. tenella* sporozoites were determined and are summarized in Table 2. The NADH-oxidase specific activity of 5.9 nmol/min/mg protein was confirmed in the mitochondria-rich fraction from *E. tenella* sporozoites, which was completely inhibited by the addition of 2 mM KCN, an inhibitor of complex IV. Consecutive addition of fumarate restored the NADH oxidation activity to 1.3 nmol/min/mg protein, and following the addition of 5 mM malonate, this activity was again abolished. This indicates that *E. tenella* possesses an intrinsic capacity to re-route the ETC dehydrogenase electron flux from oxygen to fumarate via reverse reaction of complex II, when the electron flux to oxygen is compromised. Consistently, by LC-MS (m/z 741.56 [M + Na]^+^) and HPLC-PDA analyses, we could identify a menaquinone specie, MK-8(H_2_), from the extracts of *E. tenella* sporozoites (Figures S6 and S7). Menaquinones are usually required for anaerobic respiration, such as fumarate respiration, in bacteria living under anaerobic conditions [37]. Moreover, all ETC enzyme activity deduced from the *E. tenella* genome could be determined (Table 2) either by cytochrome *c*-linked (via complex III) or by direct detection of dUQ reduction (Table 3). Amongst the five ETC dehydrogenases, NDH-2 showed the highest activity, followed by G3PDH/MQO, SDH, and DHODH (Table 3).

### 3.5. Inhibition Studies of Eimeria tenella Mitochondrial Complex II

We have previous reported that the sensitivity of complex II to classical inhibitors can vary between species [25,26,27,38]. To evaluate the sensitivity of *E. tenella* complex II to inhibitors, we tested the inhibitory effect of classical complex II inhibitors, such as atpenin A5, siccanin, carboxin, and flutolanil. Surprisingly, none of the compounds tested showed significant inhibition of complex II-III activity, except for siccanin, which showed an IC_50_ of 4.0 μM (Table 4). Siccanin was previously identified as inhibitor of complex II from fungi, *Pseudomonas aeruginosa*, rat, *L. tarentolae*, *L. major*, *Trypanosoma brucei*, and *T. cruzi* [25,26]. Although siccanin inhibited *E. tenella* complex II-III activity, it did not inhibit the SQR activity of complex II (Table 4), indicating that the primary target of siccanin in *E. tenella* is complex III rather than complex II. 

### 3.6. HrCNE and Nano-LC MS/MS Analyses of Eimeria tenella Mitochondrial Complex II

**Figure 2:**
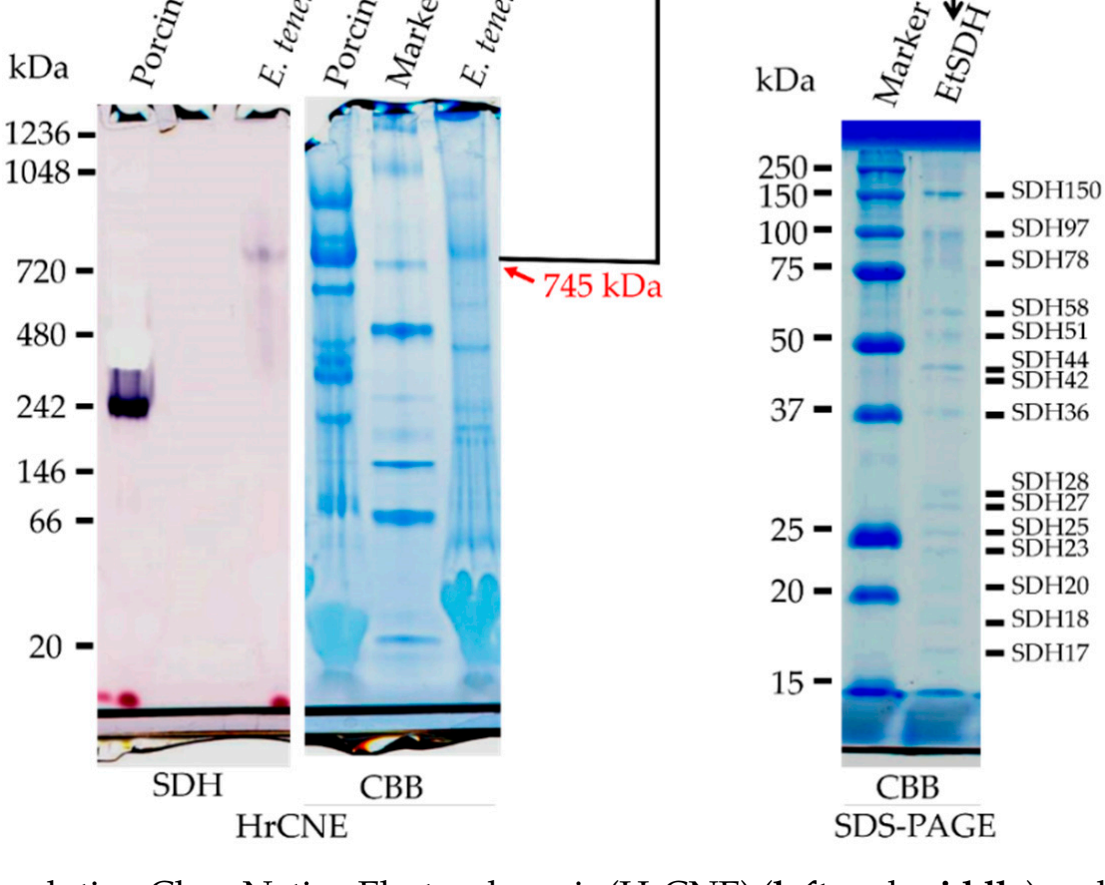
High Resolution Clear Native Electrophoresis (HrCNE) (**left** and **middle**) and two-dimensional polyacrylamide gel electrophoresis (2D-SDS-PAGE) (**right**) analysis of *E. tenella* complex II. For HrCNE gels, 10 μg and 27 μg per lane of bovine heart mitochondria and *E. tenella* mitochondria-rich fraction were loaded, respectively. Left and middle panels were stained by SDH activity and CBB staining, respectively. The red arrow indicates the location of SDH stained bands of 745 kDa in the CBB stained gel, which was cut off and loaded into 2D-SDS-PAGE gel (**right**) and stained by CBB. A total of 15 bands, named according to their calculated molecular weight, were stoichiometrically stained by CBB (**right**).

Due to the unusual property of *E. tenella* complex II, we further conducted analysis by HrCNE. As shown in Figure 2, the HrCNE followed by SDH activity staining revealed a single band corresponding to the molecular weight of 745 kDa. As a control, bovine mitochondria was included and showed a band of about 240 kDa, which was consistent to a previous report (Figure 2) [26]. Moreover, the band corresponding to *E. tenella* complex II was excised from HrCNE gel and loaded onto 2-D SDS-PAGE. According to the result shown in Figure 2, 15 bands (SDH17 to SDH150, with numbers indicating the calculated molecular weight), stoichiometric stained by CBB, were identified from the SDH activity stained band. Additionally, the band corresponding to SDH from HrCNE was also sent for nano-LC-MS/MS analysis. Among the SDH subunits, we could successfully identify the Fp (flavoprotein) and a possible candidate for the Ip (iron-sulfur protein) subunits in the SDH activity stained band (Table S1). In addition, several subunits from complex III (ubiquinol:cytochrome *c* reductase), as well as complex IV (Cytochrome *c* oxidase) and cytochrome *c* itself, were identified in the SDH activity stained band (Table S1).

## 4. Discussion

This study provides a reproducible method for the preparation of mitochondria-rich fraction from *E. tenella* sporozoites. Using this method, a high specific activity of ETC enzymes was observed in our preparations and characterized for the first time. According to our TEM analysis, the tubular mitochondria from sporozoites contains tubular cristae and is localized in the vicinity of the refractile body. Since the curvature of the cristae membrane is driven by the adenosine triphosphate (ATP) synthase [40], our result indicates the presence of an active ETC and oxidative phosphorylation, which is consistent with the high ETC activity observed in our study. In the case of the *P. falciparum* and *P. berghei* asexual blood stage, the cristae are not found in their mitochondria, whilst in the gametocytes, the presence of cristae becomes apparent [41,42]. Interestingly, the MitoTracker^®^ (Thermo Fisher Scientific) stained mitochondria were observed in close vicinity to the refractile body. The physiological function of the refractile body is still unclear, but it has been found in *Eimeria* and *Lankesterella* [43]. Because of the close location of mitochondria to the refractile body in *E. tenella* sporozoites, it is tempting to speculate that the overlapping spatial distribution of mitochondria and the refractive body might be important for their respective functions.

Previously, a cell disruption method using Teflon pestle tissue homogenizer was reported [44]. However, due to the in situ heating causing a loss of ETC enzyme activities, the longer time needed, and problems with reproducibility, this method was not tested in this study. Instead, we tested the Polytron homogenizer, which is a mild cell disruption method. We have previously reported a fast and successful disruption of *P. falciparum* and *P. berghei* by the N_2_ cavitation method [45,46,47], and in this study, we have compared the two methods in terms of the yield of ETC enzyme activity (NADH- and succinate-cytochrome *c* reductase) in a mitochondria-rich fraction obtained from *E. tenella* sporozoites. Although *Plasmodium* parasites at the erythrocytic stage could be disrupted by N_2_ cavitation at 1200 psi, *E. tenella* sporozoites could not be disrupted at the same condition, but it required a higher pressure of 2000 psi. This high pressure might be needed to break the specific membrane structure of the sporozoites due to its ability to survive in the harsh environment of the chick intestine, where the parasites are exposed to digestive enzymes. At least for NADH- and succinate-cytochrome *c* reductase activities, we have found that storage of mitochondria-rich fraction at −80 °C maintained the specific activity by 74% and 87%, respectively, at least for three days of storage.

The enrichment of mitochondria is evident due to the extremely high specific activities of NDH-2, and Complex II and III activities in our preparations. The NADH-cytochrome *c* reductase activity from our preparations ranged from 167 to 341 nmol/min/mg, which is over 10-fold higher than the reported Plasmodium mitochondria-rich preparations [39]. Similarly, complex II [41,48,49] and MQO [14] specific activities from our preparations exceeded the reported ones from Plasmodium. Although the specific activities of ETC enzymes indicate an enrichment of the mitochondria fraction, microneme and rhoptory proteins were detected by nano LC-MS/MS analysis in the SDH activity stained band by HrCNE (Table S1). This suggests that those organelles are present in the mitochondria-rich fraction used in this study.

The biochemical features of the mitochondrial energy metabolic pathway related to ETC in *E. tenella* has not been fully clarified. Previously, the mRNA expressions for most enzymes in the metabolic pathway, including glycolysis and the TCA cycle, have been reported at the extracellular stages of *E. tenella* [19], and mitochondria was confirmed in the *E. tenella* sporozoites with tubular cristae [50]. Biochemical analysis of the mitochondria-rich fraction revealed that the *E. tenella* sporozoites have an active ETC, characterized by high NDH-2 activity, followed by MQO, G3PDH, SDH, and DHODH activities. The presence of NDH-2, SDH, and MQO indicates that at the sporozoite stage, the parasites possess an active TCA cycle. In addition, G3PDH activity indicates the presence of an active glycerol metabolism and perhaps gluconeogenesis. In contrast to other ETC dehydrogenases, the DHODH activity in sporozoites was found to be the lowest (0–1.7 nmol/min/mg). Since DHODH is the rate-limiting step in pyrimidine de novo biosynthesis required for DNA and RNA synthesis, low DHODH activity is characteristic of non-dividing cells or cells at the resting state [51,52]. This is consistent with the fact that sporozoites obtained from mature oocysts are in the resting state and thus, the pyrimidine de novo biosynthesis pathway must be suppressed.

Because the oocysts sporulate under aerobic conditions and can use stored mannitol or amylopectin as carbon sources [53,54], it is possible that the parasites need an active TCA cycle and oxidative phosphorylation for effective generation of ATP, since those internal carbon sources are limited. The mitochondrial complex II can function as succinate ubiquinone reductase (SQR) that catalyzes the oxidation of succinate and supplies electrons to the quinone pool, or as quinol fumarate reductase (QFR) by catalyzing the reverse reaction of SQR [6]. Complex II working as QFR becomes important under an environment of low oxygen, where the electron flux to oxygen through complexes III and IV are not functional. Such conditions cause an accumulation of respiratory quinols and, consequently, inhibition of all upstream ETC dehydrogenases. In order to re-oxidize quinols, complex II starts to function as QFR in a process called fumarate respiration, which is found in bacteria [55], helminthes [9,56], and also in some types of cancer cells living under a tumor microenvironment [11,30,36,57,58]. One of the important findings in the current study is that NADH started to re-oxidize by the addition of fumarate once the oxidation through complex III and IV was inhibited by cyanide. This fumarate-dependent NADH re-oxidation was completely inhibited by addition of malonate, a specific inhibitor of complex II. This means that *E. tenella* sporozoites has an intrinsic capacity of fumarate respiration in addition to classical oxygen respiration, which is supported by the presence of MK-8(H_2_), a menaquinone specie (Figures S6 and S8). Such versatile mitochondrial respiration may become essential for adaptation to a hostile host intestinal environment. The presence of fumarate respiration in apicomplexan parasites has also been suggested for *P. falciparum* and *P. berghei* [59], which is consistent with our results.

We have previously reported that *P. y. yoelii* complex II was about 600-fold less sensitive to atpenin A5 [39], the most potent complex II inhibitor described to date [24]. To our surprise, complex II from *E. tenella* was found to be even more insensitive to atpenin A5. In addition, other known inhibitors of complex II tested in this study, which were siccanin [25], carboxin [38], and flutolanil [7,27], did not inhibit *E. tenella* complex II, even at higher concentration of 50 μM (Table 4). To our knowledge, *E. tenella* complex II is the most inhibitor-insensitive among this enzyme family reported so far.

Depending on the species, the composition of complex II subunits can be diverse. In general, complex II is composed by four subunits: Flavoprotein (Fp), iron-sulfur cluster (Ip), cytochrome *b* large (Cy*b*L), and small (Cy*b*S) subunits. However, complex II that physiologically functions as QFR is composed by three or four subunits and can be found in some bacteria and helminths, respectively [6]. Additionally, complex II formed by eight and 12 subunits was reported in plants [60] and trypanosomatid parasites, respectively [26,38]. Surprisingly, the *E. tenella* complex II analysed by HrCNE showed a single band of about 745 kDa stained by the in-gel SDH-activity. In the following 2D-SDS-PAGE, a total of 15 bands stoichiometrically stained by CBB could be identified from SDH-activity stained band. The possibility of a trypanosomatid-type mega size complex II in *E. tenella* was ruled out because several complex III and IV subunits were identified in an SDH-stained band by nano-LC MS/MS analysis. Instead, our data suggest that the SDH-stained band has the potential to be a respiratory supercomplex formed by complexes II, III, and IV, which has never been reported.

## 5. Conclusions

Our study provides the first evidence of an active and functional mitochondrial ETC in *E. tenella* parasites, which was only possible by the development of a new method for the isolation of mitochondria-rich fraction. Amongst the ETC enzymes, complex II showed unique biochemical features of the (i) ability to support fumarate respiration; (ii) being insensitive to practically all complex II inhibitors reported to date, and (iii) a possible formation of a respiratory supercomplex with complexes III and IV. Moreover, the differences in sensitivity to complex II inhibitors indicate that the development of *E. tenella* specific complex II inhibitor can be achieved and developed to a new generation of anticoccidian drugs with novel mechanism of action. 

## Figures and Tables

**Table 1:** Stability of NADH-cyt *c* and succinate-cyt *c* activities (Abs/min/μL).

	NADH-cyt *c*	Succinate-cyt *c*
Day 0-Polytron	0.011 (100%)	0.004 (100%)
Day 0-N_2_ Cavitation	0.050 (100%)	0.010 (100%)
Day 3–4 °C-Polytron	0.006 (57%)	0.003 (63%)
Day 3–4 °C-N_2_ Cavitation	0.008 (16%)	0.007 (69%)
Day 3–80 °C-Polytron	0.011 (100%)	0.026 (65%)
Day 3–80 °C-N_2_ Cavitation	0.037 (74%)	0.009 (87%)

Data from *E. tenella* mitochondria prepared by two different methods (Polytron or N_2_ cavitation), at day 0 and day 3 stored at 4 °C or −80 °C. The number in parenthesis represents the percentage activity relative to day 0. NADH: nicotinamide adenine dinucleotide.

**Table 2:** Reproducibility of mitochondria preparation from *E. tenella* sporozoites.

	Specific Activity (nmol/min/mg)			
	A	B	C	D
NADH-cyt *c* ^1^	341 ± 18	228 ± 16	167 ± 8	189 ± 28
G3P-cyt *c* ^1^	137 ± 3	94 ± 1	101 ± 6	119 ± 5
Malate-cyt *c* ^1^	108 ± 9	75 ± 9	95 ± 5	77 ± 9
Succinate-cyt *c* ^1^	50 ± 7	50 ± 7	71 ± 2	54 ± 2
Dihydroorotate-cyt *c* ^1^	0.0	0.0	1.7	1.7

^1^ Data represent the average of cytochrome *c*-linked specific activity measured in triplicates, except for dihydroorotate-cyt *c* activity, by four independent experiment (A, B, C, and D) performed at different dates.

**Table 3:** Specific activities of *E. tenella* electron transport chain dehydrogenases.

	Specific Activity ^1^
NADH oxidase	5.9 ± 0.9
+ 2 mM KCN	0.0 ± 0.3
+ 2 mM fumarate	1.3 ± 0.3
+ 5 mM malonate	−0.5 ± 0.3
NADH-cyt *c*	167 ± 8
NADH-dUQ	224 ± 19
G3P-cyt *c*	101 ± 6
G3P-dUQ	61 ± 2
Malate-cyt *c*	95 ± 5
Malate-dUQ	67 ± 3
Succinate-cyt *c*	71 ± 2
Succinate-dUQ	25 ± 2
Dihydroorotate-cyt *c*	1.7

^1^ Data represent the average of specific activity (μmol/min/mg protein) measured in triplicates from experiment C (Table 2), except for dihydroorotate-cyt *c* activity.

**Table 4:** Insensitivity of *E. tenella* complex II to classical inhibitors.

	IC_50_ of Succinate-cyt *c* Activity (μM)			
	*E. tenella*	*P. y. yoelii* [39]	Rat	Porcine
Atpenin A5	>50	4.2 ± 0.2 [39]	0.007 ± 0.0003 [39]	N.D.
Siccanin	4.0 ± 0.6 (>50) ^1^	N.D. [39]	9.3 ± 1.0 [25]	861 ± 822 [25]
Carboxin	>50	3.6 ± 1.0 [39]	3.8 ± 0.1 [39]	N.D.
Flutolanil	>50	>100 [39]	>100 [39]	>50 [7]
Stigmatellin	78.4 ± 13.1% ^2^	N.D.	N.D.	N.D.

^1^ Value in parenthesis is the IC_50_ determined for succinate-dUQ activity. ^2^ Percentage inhibition at 100 nM concentration of stigmatellin, which was used as a positive control for complex III inhibition. N.D.: Not determined.

## Supplementary Materials

The following are available online at http://www.mdpi.com/2073-4425/10/1/29/s1, Table S1: Nano-LC MS/MS analysis of SDH activity stained band from HrCNE. Figures S1, S2, S3, S4 and S5: Alignment and percentage identity analysis of NDH-2, SDH1, SDH2, MQO and G3PDH, respectively. Figures S6 and S7: Quinone content analysis from *E. tenella* sporozoites by HPLC-PDA and LC-MS, respectively.
