# Supplementary material for: Novel Characteristics of Mitochondrial Electron Transport Chain from Eimeria tenella
Source: Genes (Basel). 2019 Jan 8;10(1):29. doi: 10.3390/genes10010029 (PMC6356742; doi:10.3390/genes10010029)
Supplement: Supplementary file 1 [file genes-10-00029-s001.pdf]

Table S1. Nano-LC MS/MS analysis of SDH activity stained band from HrCNE.

	Protein	MW	Score	Peptide	Coverage	Accession	*Note**
1	Microneme protein Etmic-1	74,778	993	33	41	O43981	*gi|2707733*
2	Microneme protein 3	105,644	555	18	23	B6VCV4	*gi|211905276*
3	Mitochondrial-processing peptidase beta subunit, putative	57,496	389	22	52	U6KT33	*gi|557144991*
4	Mitochondrial-processing peptidase alpha subunit, putative	62,775	311	16	30	U6KH88	*gi|557137520*
5	Microneme protein Etmic-2 Microneme protein-2	35,087	281	11	42	U6KXC3	*gi|4164596*
6	Ubiquinol-cytochrome *c* reductase domain-containing protein, putative (Fe2S2 subunit)	43,429	215	8	32	U6KR99	*gi|557141998*
7	Putative rhoptry neck protein	232,935	196	9	5	A7DZP6	*gi|153581935*
8	Ubiquinol-cytochrome *c* reductase complex 14 kDa protein, putative	25,480	158	6	23	U6KKV7	*gi|557139144*
9	Uncharacterized protein (one transmembrane region)	23,144	147	4	31	U6KQV1	*gi|557139998*
10	Uncharacterized protein	33,398	140	9	36	U6KXU8	*gi|557147873*
11	Cytochrome *c* oxidase subunit Vb	25,120	133	36	38	C8TDR9	*gi|118490698*
12	Cytochrome *c*1, putative	46,055	116	4	14	U6L5K5	*gi|557147988*

13	Nucleoside-triphosphatase, putative	100,043	91	2	3	U6KZA4	*gi|557148408*
14	Uncharacterized protein (3 transmembrane region) *[Eimeria necatrix]*	27,173	88	5	16	U6N036	*gi|557233254*
15	Transhydrogenase	108,380	88	3	3	Q07600	*gi|305062*
16	Superoxide dismutase	24,925	87	6	37	U6KZ13	*gi|557146360*
17	Sporozoite antigen	22,369	86	2	12	P15744	*gi|113933*
18	Uncharacterized protein (one transmembrane region)	15,581	80	2	15	U6KIX2	*gi|557138220*
19	Uncharacterized protein (one transmembrane region)	8,488	72	1	18	U6KUQ3	*gi|557146721*
20	Quinol Cytochrome *c* Reductase subunit 9	12,113	64	3	36	U6KG27	*gi|557137182*
21	Uncharacterized protein (two transmembrane region)	23,540	62	1	4	U6LB54	*gi|557154558*
22	Uncharacterized protein (Iron-sulfur cluster binding domain)	13,354	61	3	22	U6KZ96	*gi|557146466*
23	Cytochrome *c* oxidase subunit II, putative	22,720	59	2	12	H9BA62	*gi|357017687*
24	Cytochrome *c* oxidase IV	23,681	56	5	24	H9B966	*gi|357016995*

25	Succinate dehydrogenase (Ubiquinone) flavoprotein subunit, mitochondrial, putative	71,427	55	1	2	U6KGY0	*gi|557137402*
26	Cytochrome c oxidase subunit 2	23,918	54	4	18	H9B9F9	*gi|357017181*
27	Uncharacterized protein (one transmembrane region)	13,843	52	4	50	U6L2Z0	*gi|557146997*
28	Zgc:110343, related (peroxyredoxin)	28,449	52	3	14	U6KYA3	*gi|557147835*

Yellow, orange and green cells are identified peptides related to complex II, III and IV, respectively.

Figure S1

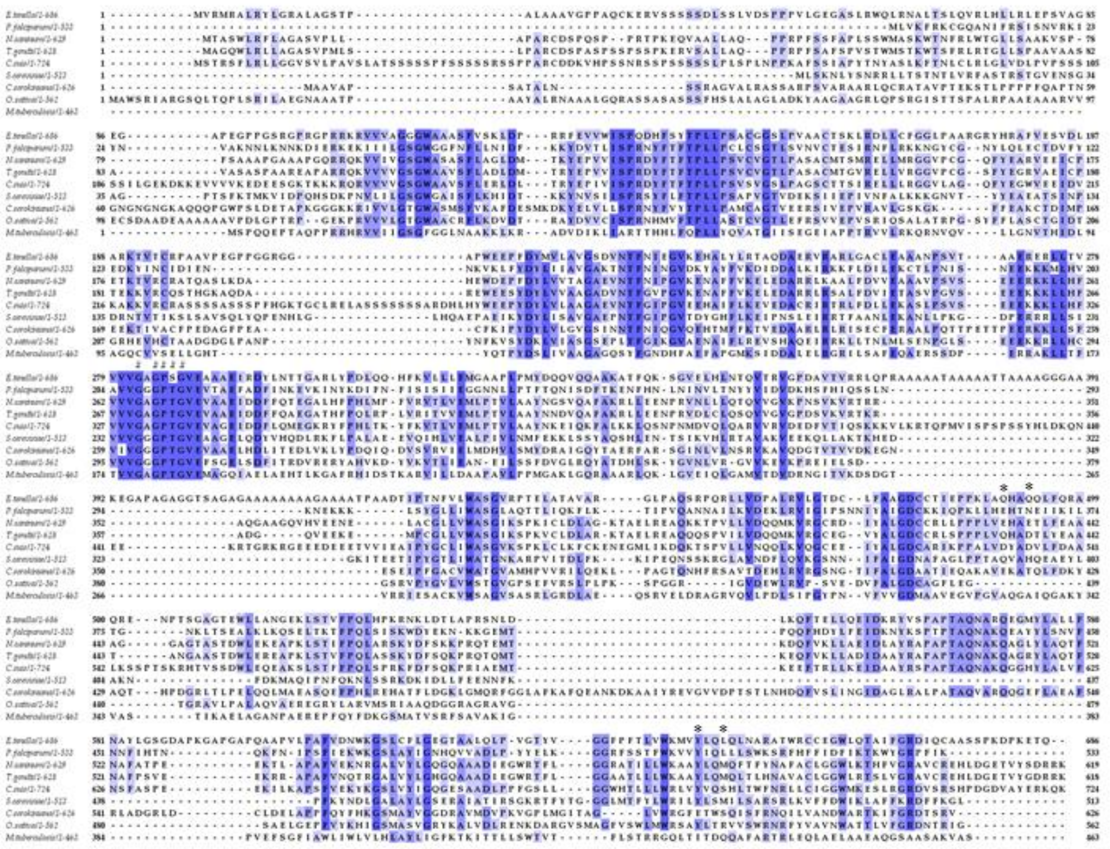

	E. tenella	*P. falciparum*	*N. caninum*	*T. gondii*	*C. suis*	*S. cerevisiae*	*C. sorokiniana*	*O. sativa*	*M. tuberculosis*
E. tenella		25.45	36.03	33.96	34.06	23.67	29.51	26.10	24.08
P. falciparum			33.39	31.57	31.34	28.37	27.01	25.05	20.25
N. caninum				76.92	48.79	29.26	30.09	26.45	23.41
T. gondii					50.28	27.44	28.95	28.08	22.92
C. suis						26.38	27.45	25.52	22.86
S. cerevisiae							29.87	31.94	26.00
C. sorokiniana								30.85	23.30
O. sativa									28.66
M. tuberculosis									

Figure S1. Top: sequence alignment of amino acid sequences of NDH2 from *E. tenella* and other organisms. Residues were colored according to percentage identity by Jalview. The conserved GXGXXG motifs as potential NADH-binding residues and the ubiquinone binding residues according to the crystal structure of *S. cerevisiae* Ndi1 (PDB: 5YJW) are shown with "#" and "*" symbols, respectively. Bottom: Amino acid sequence identity. Abbreviations; *E. tenella*: *Eimeria tenella* (XP_013228441), *P. falciparum*: *Plasmodium falciparum* (XP_001352022), *N. caninum*: *Neospora caninum* (XP_003879906), *T. gondii*: *Toxoplasma gondii* (XP_002369675), *C. suis*: *Cystoisospora suis* (PHJ19914), *S. cerevisiae*: *Saccharomyces cerevisiae* (EDN64274), *M. tuberculosis*: *Mycobacterium tuberculosis* (L7N5D1_MYCTO), *C. soroki*: *Chlorella sorokiniana* (PRW59413), *O. sativa*: *Oryza sativa* (XP_015644788).

Figure S2

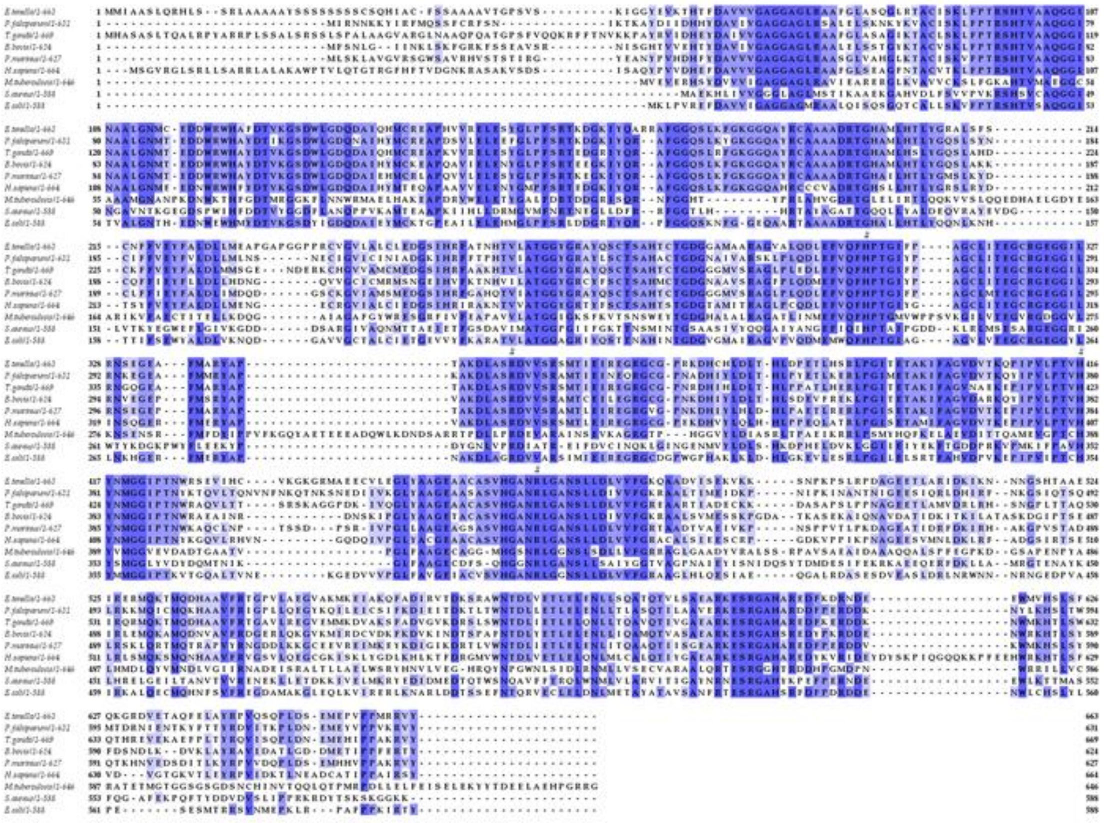

	E. tenella	*P. falciparum*	*T. gondii*	*B. bovis*	*P. marinus*	*H. sapiens*	*M. tuberculosis*	*S. aureus*	*E. coli*
E. tenella		64.59	71.41	63.33	68.54	60.56	31.76	30.58	52.37
P. falciparum			66.56	64.47	67.19	60.09	30.75	31.94	49.67
T. gondii				63.54	70.56	62.19	31.78	31.16	51.82
B. bovis					64.08	61.30	32.09	31.57	51.41
P. marinus						62.02	32.37	31.48	52.28
H. sapiens							30.51	30.29	51.22
M. tuberculosis								26.58	34.02
S. aureus									31.33
E. coli									

Figure S2. Top: sequence alignment of amino acid sequences of SDH1 (flavoprotein subunit) from *E. tenella* and other organisms. Residues were colored according to percentage identity by Jalview. Conserved residues involved in the dicarboxylate-binding, according to the crystal structure of *E. coli* succinate dehydrogenase SDH (PDB: 1NEK), are shown with "#" symbol. Bottom: Amino acid sequence identity. Abbreviations; *E. tenella*: *Eimeria tenella* (XP_013228047), *P. falciparum*: *Plasmodium falciparum* (CZT98601), *T. gondii*: *Toxoplasma gondii* (KYF42821), *B. bovis*: *Babesia bovis* (XP_001610645), *P. marinus*: *Perkinsus marinus* (EER16513), *H. sapiens*: *Homo sapiens* (AAA20683), *M. tuberculosis*: *Mycobacterium tuberculosis* (BAX25882), *S. aureus*: *Staphylococcus aureus* (EWC66604), *E. coli*: *Escherichia coli* (KGM73400).

Figure S3

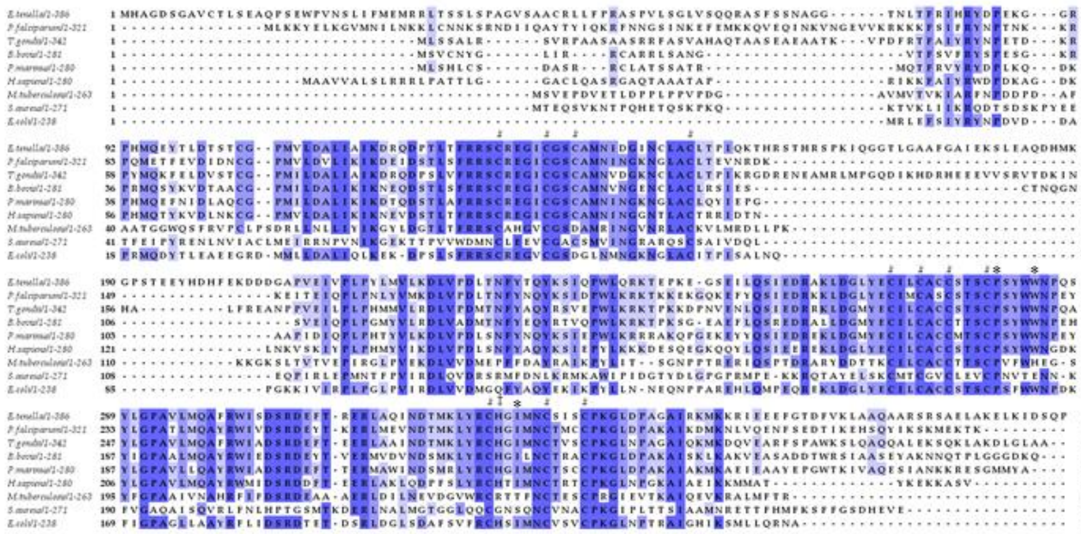

	E. tenella	*P. falciparum*	*T. gondii*	*B. bovis*	*P. marinus*	*H. sapiens*	*M. tuberculosis*	*S. aureus*	*E. coli*
E. tenella		46.34	58.12	50.15	52.28	48.17	32.47	19.64	42.11
P. falciparum			50.29	58.70	59.42	53.19	34.83	22.63	49.38
T. gondii				54.06	54.06	49.23	33.11	19.17	46.57
B. bovis					59.79	54.92	36.40	19.42	48.54
P. marinus						62.26	34.59	20.44	49.17
H. sapiens							35.58	23.51	53.11
M. tuberculosis								23.68	41.39
S. aureus									24.39
E. coli									

Figure S3. Top: sequence alignment of amino acid sequences of SDH2 (iron-sulfur cluster subunit) from *E. tenella* and other organisms. Residues were colored according to percentage identity by Jalview. Conserved residues involved in the coordination of the iron-sulfur clusters, and interactions with cytochrome *b* and ubiquinone, according to the crystal structure of *E. coli* succinate dehydrogenase SDH (PDB: 1NEK), are shown with "#", "‡" and "∗" symbols, respectively. Bottom: Amino acid sequence identity. Abbreviations; *E. tenella*: *Eimeria tenella* (XP_013233141), *P. falciparum*: *Plasmodium falciparum* (CZT99291), *T. gondii*: *Toxoplasma gondii* (CEL77101), *B. bovis*: *Babesia bovis* (EDO06889), *P. marinus*: *Perkinsus marinus* (EEQ98108), *H. sapiens*: *Homo sapiens* (AAA81167), *M. tuberculosis*: *Mycobacterium tuberculosis* (KLL09309), *S. aureus*: *Staphylococcus aureus* (AKJ17007), *E. coli*: *Escherichia coli* (KIH34397).

Figure S4

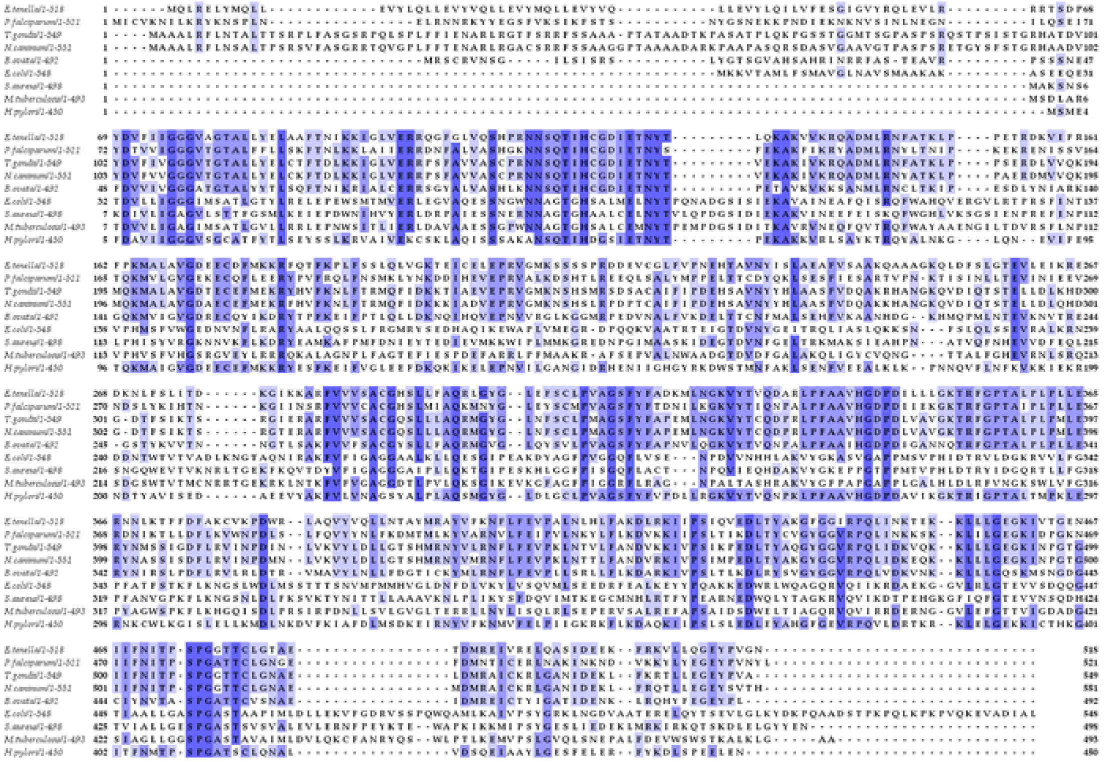

	E. tenella	*P. falciparum*	*T. gondii*	*N. caninum*	*B. ovata*	*E. coli*	*S. aureus*	*M. tuberculosis*	*H. pylori*
E. tenella		48.85	59.19	58.38	48.89	20.04	20.16	22.79	42.95
P. falciparum			50.38	49.52	50.91	21.55	21.60	22.81	42.73
T. gondii				87.64	49.80	22.54	21.27	23.87	42.73
N. caninum					49.80	19.65	20.45	24.80	42.51
B. ovata						22.61	22.13	23.55	44.25
E. coli							45.40	47.78	22.84
S. aureus								41.53	21.81
M. tuberculosis									22.29
H. pylori									

Figure S4. Top: sequence alignment of amino acid sequences of MQO from *E. tenella* and other organisms. Residues were colored according to percentage identity by Jalview. Bottom: Amino acid sequence identity. Abbreviations; *E. tenella*: *Eimeria tenella* (CAK51433), *P. falciparum*: *Plasmodium falciparum* (CAG25406), *T. gondii*: *Toxoplasma gondii* (ABE76504), *N. caninum*: *Neospora caninum* (XP_003881055), *B. ovata*: *Babesia ovata* (GBE62760), *E. coli*: *Escherichia coli* (OAF94253), *S. aureus*: *Staphylococcus aureus* (ABR53505), *M. tuberculosis*: *Mycobacterium tuberculosis* (KXN95083), *H. pylori*: *Helicobacter pylori* (HP_0086).

Figure S5

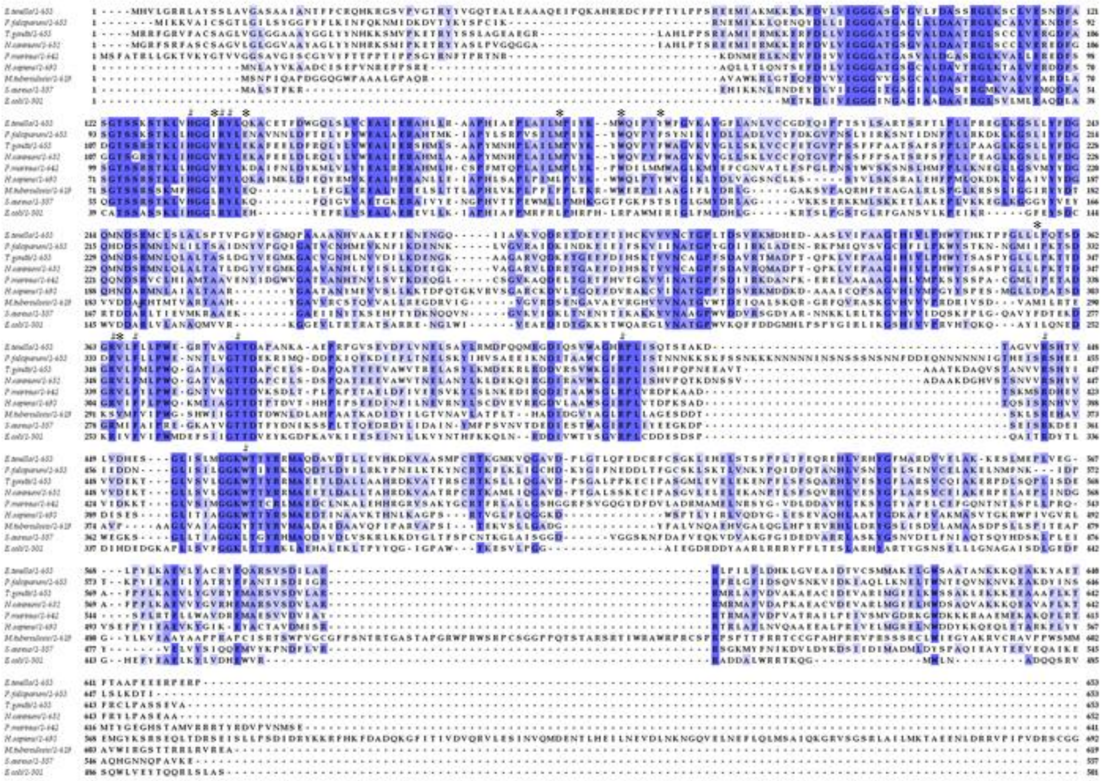

	E. tenella	*P. falciparum*	*T. gondii*	*N. caninum*	*P. marinus*	*H. sapiens*	*M. tuberculosis*	*S. aureus*	*E. coli*
E. tenella		37.35	52.69	52.91	37.42	39.90	29.04	30.22	28.10
P. falciparum			40.27	39.85	35.42	35.20	25.71	29.01	25.73
T. gondii				83.44	39.45	41.15	30.23	28.78	30.74
N. caninum					41.64	41.60	30.69	27.60	28.98
P. marinus						40.00	29.66	28.50	27.55
H. sapiens							31.29	31.26	29.96
M. tuberculosis								30.07	31.01
S. aureus									30.90
E. coli									

Figure S5. Top: sequence alignment of amino acid sequences of G3PDH from *E. tenella* and other organisms. Residues were colored according to percentage identity by Jalview. Conserved residues possibly involved in the binding of glycerol-3-phosphate and ubiquinone, according to the crystal structure of *E. coli* G3PDH (PDB: 2R46), are shown with "#" and "∗" symbols, respectively. Bottom: Amino acid sequence identity. Abbreviations; *E. tenella*: *Eimeria tenella* (XP_013232618), *P. falciparum*: *Plasmodium falciparum* (PKC45437), *T. gondii*: *Toxoplasma gondii* (EPR62522), *N. caninum*: *Neospora caninum* (CEL66611), *P. marinus*: *Perkinsus marinus* (XP_002774011), *H. sapiens*: *Homo sapiens* (XP_011509280), *M. tuberculosis*: *Mycobacterium tuberculosis* (CKM72788), *S. aureus*: *Staphylococcus aureus* (KII21235), *E. coli*: *Escherichia coli* (WP_021514332).

Figure S6

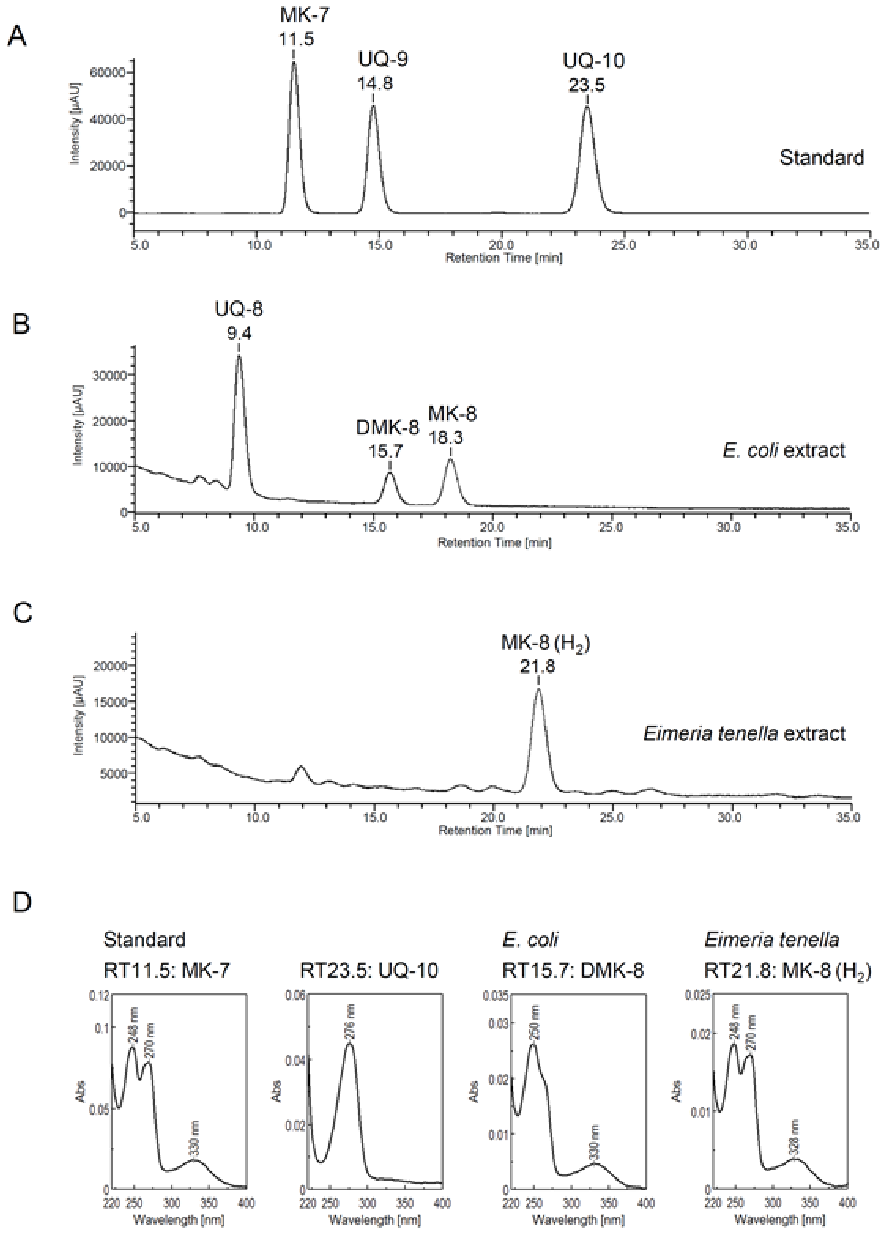

Figure S6. Quinone composition of *E. tenella* extract analysed by HPLC-PDA. **(a)**, mixture of standard quinones, MK-7, UQ-9 and UQ-10. **(b)**, *E. coli* extract containing UQ-8, DMK-8 and MK-8. **(c)**, *E. tenella* extract. **(d)**, UV-spectra of MK-7, UQ-10, DMK-8 and MK-8(H_2).

Figure S7

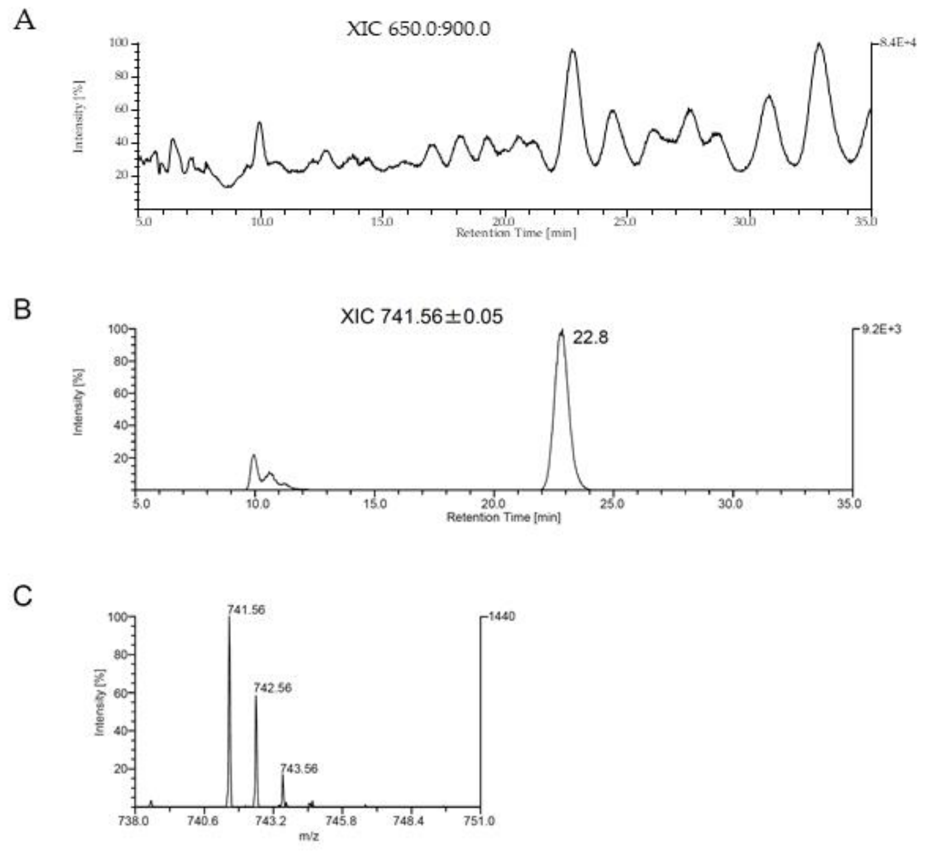

Figure S7. LC-MS analysis of *E. tenella* extract. **(a)**, extracted ion chromatogram (XIC) of m/z range 650-900. **(b)**, XIC of m/z 741.56 ± 0.05 corresponding to $[M + Na]^+$ from MK-8(H_2). **(c)**, Mass spectrum of the component eluted at retention time 22.8 minutes.
